# Cell reproductive patterns in the green alga *Pseudokirchneriella subcapitata* (=*Selenastrum capricornutum*) and their variations under exposure to the typical toxicants potassium dichromate and 3,5-DCP

**DOI:** 10.1371/journal.pone.0171259

**Published:** 2017-02-02

**Authors:** Takahiro Yamagishi, Haruyo Yamaguchi, Shigekatsu Suzuki, Yoshifumi Horie, Norihisa Tatarazako

**Affiliations:** 1 Ecotoxicity Reference Laboratory, Risk Assessment Science Collaboration Office, Center for Health and Environmental Risk Research, National Institute for Environmental Studies, Tsukuba, Ibaraki, Japan; 2 Center for Environmental Biology and Ecosystem Studies, National Institute for Environmental Studies, Tsukuba, Ibaraki, Japan; University of Massachusetts Amherst, UNITED STATES

## Abstract

*Pseudokirchneriella subcapitata* is a sickle-shaped freshwater green microalga that is normally found in unicellular form. Currently, it is the best known and most frequently used species of ecotoxicological bioindicator because of its high growth rate and sensitivity to toxicants. However, despite this organism’s, our knowledge of its cell biology—for example, the patterns of nuclear and cytoplasmic division in the mitotic stage—is limited. Although it has been reported that *P*. *subcapitata* proliferates by popularity forming four daughter cells (autospores) through multiple fission after two nuclear divisions, here, we report two additional reproductive patterns by which two autospores are formed by binary fission (“two-autospore type”) and eight autospores are formed by multiple fission (“eight-autospore type”). Moreover, we found that cell reproductive patterns differed markedly with the culture conditions or with exposure to either of two typical toxicants, potassium dichromate (K_2_Cr_2_O_7_) and 3,5-dichlorophenol (3,5-DCP). The eight-autospore type occurred at the highest frequency in the early phase of culture, but it disappeared under 3,5-DCP at 2.0 mg/L. Under 0.3 mg/L K_2_CrO_7_ (Cr(VI)) the eight-autospore type took substantially longer to appear than in control culture. The two-autospore type occurred only in the late phase of culture. To our knowledge, this is the first detailed evaluation of the reproductive patterns of *P*. *subcapitata*, which changed dramatically in the presence of toxicants. These findings suggest that observation of the reproductive patterns of *P*. *subcapitata* will help to elucidate different cell reactions to toxicants.

## Introduction

In water-quality monitoring and ecological risk assessment, bioassays are deployed as valuable tools to reveal the biological effects of chemicals or wastewater. Chemical analyses are also important for identifying compounds present in the environment, but they do not give us insights into the bioavailability of known toxicants. Currently, protocols for ecotoxicologic tests using a variety of aquatic organisms have been published by organizations such as the International Organization for Standardization, the Organization for Economic Cooperation and Development (OECD), and the United States Environmental Protection Agency.

*Pseudokirchneriella subcapitata* (Korshikov) F.Hindák, known as *Selenastrum capricornutum* Printz or *Raphidocelis subcapitata* (Korshikov) Nygaard, Komárek, J.Kristiansen & O.M.Skulberg, is a sickle-shaped, freshwater green microalga that is normally found in unicellular form [1–3]. Currently, it is the best known and most frequently used ecotoxicological bioindicator species because of its high growth rate, sensitivity to toxicants, and good reproducibility compared with those of other algae. However, despite this organism’s popularity, our knowledge of its cell biology—such as the patterns of nuclear and cytoplasmic division in the mitotic stage—is limited. Machado and Soares [4], by using SYTOX Green to visualize nuclei, found that it proliferates by forming four daughter cells (autospores) through multiple fission after two nuclear divisions. Subsequently, the four autospores are typically released through rupture of the parental cell wall without forming colonies. However, it is unclear how cytokinesis is achieved in the multinucleated cells containing four nuclei before autospore release. Machado and Soares [4] also showed that the presence of metals (Cd, Cr, Cu, or Zn) can modify algal cell volume, as previously reported [5]. Although similar cell-size changes have been reported in other algae [6–10], their mechanism is unclear.

*Scenedesmus*, a green microalgae closely related to *Pseudokirchneriella*, differs from *P*. *subcapitata* by the formation of coenobia in the first. Unlike in *P*. *subcapitata*, in *Scenedesmus* the patterns of nuclear and cytoplasmic division in the mitotic stage have been relatively well studied. *Scenedesmus* forms four or eight daughter cells by multiple fission after multiple nuclear division [11]. In the field, the daughter cells typically link to each other and form a colony enclosed by the parental cell wall. However, *Scenedesmus* seldom produces typical colonies in the laboratory [12]. Numerous factors, such as the release of chemicals from active grazers, as well as nutrient uptake and light, have been suggested to affect colony formation in *Scenedesmus* [12–15].

Here, we report two additional cell reproductive patterns in *P*. *subcapitata* apart from that by which four autospores are formed. In addition, we evaluated the frequency distributions of the different cell reproductive patterns from 24 to 120 h after the initial exposure to potassium dichromate (K_2_Cr_2_O_7_) or 3,5-dichlorophenol (3,5-DCP), both of which are commonly used as toxicants in interlaboratory comparisons of algal toxicity testing [16]. Finally, we discuss the relationship between cell size and cell reproduction patterns in *P*. *subcapitata*.

## Materials and methods

### Culture

An axenic culture of *Pseudokirchneriella subcapitata* acquired from the Micro Culture Collection of the National Institute of Environmental Studies (NIES), Tskuka Japan. The strain is NIES-35, and is a duplicate of NIVA-CHL1 [1], also listed in other collections: the Culture Collection of Algae and Protozoa (CCAP 278/4), the UTEX Culture Collection of Algae at the University of Texas at Austin (UTEX1648), the American Type Culture Collection (ATCC 22662), the University of Toronto Culture Collection of Algae and Cyanobacteria (UTCC 37), and the Collection of Algae at the University of Göttingen (SAG 61.81). The culture was maintained at 23 ± 1°C under continuous illumination by white fluorescent light (photosynthetically active radiation 60 to 80 μmol·m^−2^s^−1^) with orbital agitation (100 rpm) in OECD test medium [17]. A preculture of algae was started at least 6 days before the beginning of the test performed in OECD medium. For the main experiment, algae were harvested from the batch culture during their exponential growth phase.

*P*. *subcapitata* is currently regarded as a synonym of *Raphidocelis subcapitata*, and *R*. *subcapitata* is currently accepted name for the type species [1–3], therefore, the use of *R*. *subcapitata* is most appropriate for this species. However, considering that in ecotoxicology the use of *P*. *subcapitata* more general than *R*. *subcapitata*, we chose the use of *P*. *subcapitata* for this species in this study.

### Phylogenetic analysis of plastid-encoded Rubisco large subunit

Total DNA of *Pseudokirchneriella subcapitata* (NIES-35) was extracted using DNeasy Plant Mini Kit (Qiagen, Hilden, Germany) following manufacture’s protocol. The DNA was fragmented to approximately 550-bp segments using a Covaris M220 ultrasonicator (Covaris, Woburn, MA, USA). The genomic library was constructed using a TruSeq Nano DNA library prep kit for NeoPrep (Illumina, San Diego, CA, USA) and sequenced by the MiSeq platform (Illumina) using the 600-cycle MiSeq reagent kit version 3. Low-quality reads/bases were filtered using Trimmomatic version 0.36 [18]. *De novo* assembly was performed using SPAdes 3.9.0 [19]. A sequence of plastid-encoded Rubisco large subunit (*rbcL*) was obtained and deposited on DNA Data Bank of Japan (DDBJ) under the accession number LC200423. Basic Local Alignment Search Tool (Blast) [20] was used to verify the identity of the strain NIES 35. The sequences of *rbcL* were aligned using MUSCLE [21] integrated into MEGA7 [22]. Nucleotide substitution model selection and construction of a maximum likelihood tree were carried out by using MEGA7 [22]. GTR+G model was chosen in this analysis. Bootstrap values were calculated with 1,000 pseudoreplicates.

### Fluorescence microscopy of nuclei stained with DAPI

Cells were fixed for 15 min at room temperature with 4% formaldehyde (Wako Pure Chemical Industries, Ltd., Osaka, Japan) in PBS buffer (137 mM NaCl, 8.10 mM NaHPO_4_, 2.68 mM KCl, 1.47 mM KH_2_PO_4_; pH 7.4). After three rinses with TBS buffer (20 mM Tris, 150 mM NaCl; pH 7.4) and centrifugation (3 min at 5000*g*), each sample was stained with DAPI (Sigma-Aldrich, St. Louis, MO, USA, 1 μg·mL^–1^ in TBS) for 30 min at room temperature. After three rinses with TBS and centrifugation (3 min at 5000*g*), the sample was mounted in 50% glycerol—TBS containing 0.1% *p*-phenylendiamine and observed under an Olympus BX51 epifluorescence microscope (Olympus Optical Co., Tokyo, Japan). At least 100 cells were observed in each of three independent experiments.

### Growth inhibition tests and measurement of the frequency distributions of cells in different reproduction patterns

Growth inhibition tests were performed according to OECD Test Guideline No. 201 [17]. We prepared 100-mL algal suspensions containing the test chemicals in 300-mL Erlenmeyer flasks, with an initial algal concentration of 5 × 10^3^ cells mL^–1^. Five replicate tests for each concentration were performed. The flasks were continuously shaken with an orbital agitation of 100 rpm at 23 ± 1°C under white fluorescent light (60 to 80 μmol·m^−2^s^−1^). For DAPI staining, we collected 2 to 10 mL of the suspension every 24 h for up to 120 h after the start of exposure to each toxicant, adding to 1 mL of the suspension to measure algal cell density and size. Algal cell density and size were measured with a Sysmex CDA-500 electronic particle counter (Sysmex, Kobe, Japan). Two chemicals that are commonly used as test toxicants were used: K_2_Cr_2_O_7_ (Lot HWP7907, >99.5%) and 3,5-DCP (an inhibitor of respiration; Lot: SDK3769, >98.0%). The 3,5-DCP was dissolved in OECD medium, using N,N-dimethylformamide (DMF) as a vehicle. The final concentration of DMF in the algal suspension was less than 100 ml/L, according to OECD TG201 [17]. These chemicals were acquired from Wako Pure Chemical Industries. To measure the frequencies of cells (with one, two, four, or eight nuclei) in the different reproduction patterns under each toxicant, the collected samples were stained with DAPI as described above. At least 100 cells were counted in each experiment. Data are the means of at least 100 cells in each of five independent experiments.

## Results

**Fig 1:**
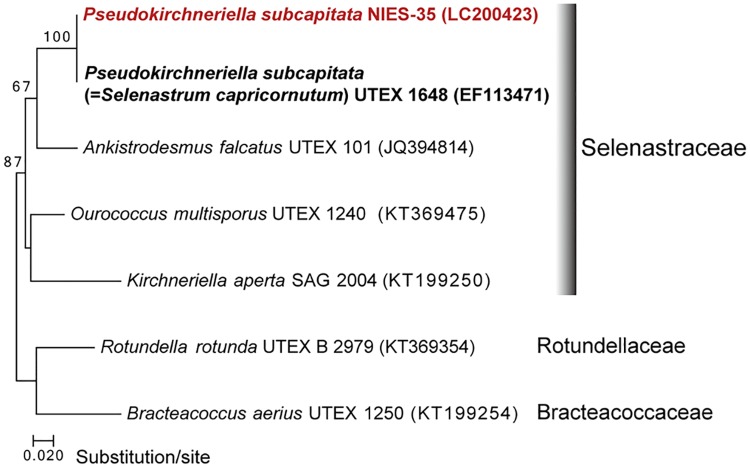
Maximum likelihood (ML) tree based on the plastid-encoded Rubisco large subunit (*rbcL*). Numbers on branches indicate bootstrap values (1,000 replicates) from ML analysis. Only bootstrap values >50% are shown.

*Pseudokirchneriella subcapitata* is commonly used in toxicity tests worldwide. *P*. *subcapitata* used in this study (NIES-35) is a duplication of NIVA-CHL1 [1], and is the same strain as ATCC 22662 and UTEX 1648 (= *Selenastrum capricornutum*), and so on. Just in case, in order to ensure identity of NIES-35 with phylogenetic analysis, we chose the sequence of plastid-encoded Rubisco large subunit (*rbcL*) because of many genes, the only *rbcL* originated from *S*. *capricornutum* (UTEX 1648) has been sequenced in high quality and available in public database. In this study, we newly determined the sequence of *rbcL* from *P*. *subcapitata* (NIES-35) using high-throughput Illumina Miseq platform. The sequence of *rbcL* from *S*. *capricornutum* (UTEX 1648), of which accession number is EF113471, was deposited in the Genbank/EMBL/DDBJ in 2006, and presumably generated by Sanger sequence platform. The sequences of *rbcL* from *P*. *subcapitata* (NIES-35) and *S*. *capricornutum* (UTEX 1648) were nearly identical with only two nucleotides substitutions. Phylogenetic analysis of *rbcL* revealed that *P*. *subcapitata* (NIES-35) and *S*. *capricornutum* (UTEX 1648) form a clade in the Selenastraceae (Fig 1). We speculate that this genetic difference between two strains are derived only by the sequence platform differences. Thus, the two strains can be identified as the same species by molecular biology and morphology.

**Fig 2:**
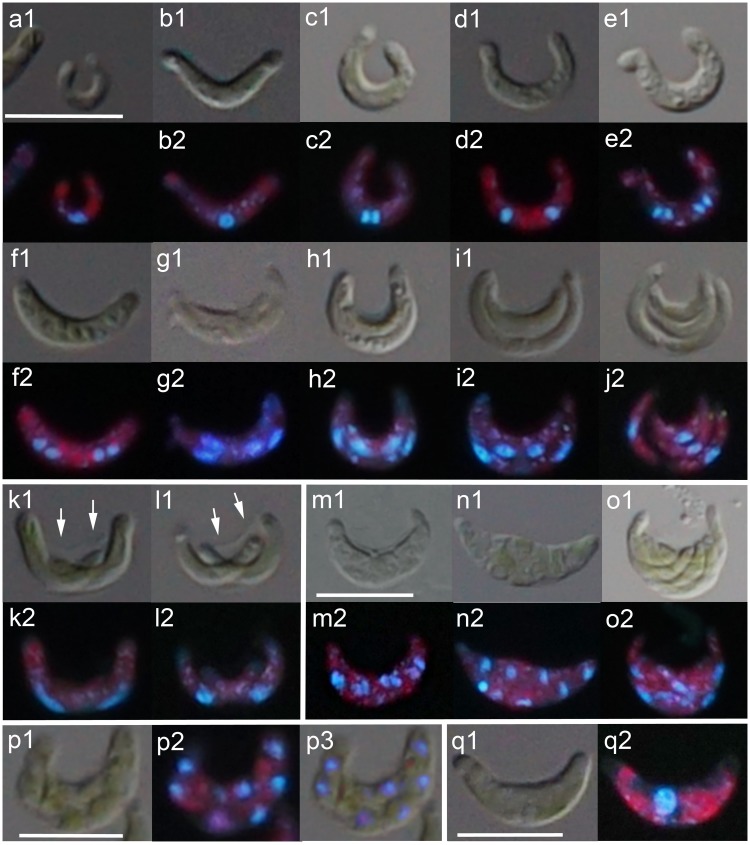
Cell reproductive patterns in *Pseudokirchneriella subcapitata*, focusing on cytoplasmic and nuclear division. Cells were observed by differential interference contrast microscopy (a1-q1) or DAPI staining (a2-q2). Three reproductive patterns were found in the population 72 h after the beginning of culture, namely the four-autospore type (a-j), the two-autospore type (k and l), and the eight-autospore type (m-o). Also shown are cells with cleavage furrows around the nuclei (p) and a cell with a single giant nucleus (q). Arrows in (k1) and (l1) indicate the wall of the parental cell. Scale bars in (a1), (m1), (p1), and (q1), 10 μm. Scale bar in (a1) applies to (b-l). Scale bar in (m1) also applies to (n and o).

To analyze the cell reproductive patterns of *P*. *subcapitata*, cells at 72 h under OECD medium were observed by staining with DAPI (Fig 2). Most of the cells divided by multiple fission after two nuclear divisions, forming four daughter cells (autospores) inside a parental cell (Fig 2a–2j). A single cell with one nucleus increased in volume (Fig 2a and 2b); this was followed by two nuclear divisions, resulting in a multinucleated cell with four nuclei (Fig 2c–2f). Cytokinesis began with a cleavage furrow along the longitudinal axis of the cell; two daughter cells were formed inside the parent cell, and each daughter cell contained two nuclei (Fig 2g–2i). Subsequent cytokinesis occurred along the transverse axis of the outside cell of the arc, whereas the inside cell divided along its longitudinal axis, resulting in the formation of four autospores inside the parental cell (Fig 2j).

In addition to this four-autospore type of reproductive pattern, we found a two-autospore type (Fig 2k and 2l) and an eight-autospore type (Fig 2m–2o). Appearance of these latter two types in the cell population was rare after 72 h in OECD medium compared with that of the four-autospore type. In the case of the two-autospore type, cytokinesis occurred along the transverse axis of the cell, and each of two daughter cells grew inside the parental cell until their release (Fig 2k and 2l). In the case of the eight-autospore type, multinucleated cells with eight nuclei were formed after three continuous nuclear divisions; their sizes were much larger than the two- or four-nucleated cells (Fig 2m and 2n). Autospore formation by repeated binary fission, as occurred in the four-autospore type, was not observed. Instead, cells with a cleavage furrow around each nucleus were observed, indicating that autospore formation in the multinucleated cells with eight nuclei began with separation of the cytoplasm into eight compartments (Fig 2o and 2p). Cells with a single giant nucleus were also observed (Fig 2q), but at low frequencies. These cells were much larger than the usual single-nucleus cells, and they appeared to have a greater DNA content than the usual single-nucleus cells (Fig 2q).

**Fig 3:**
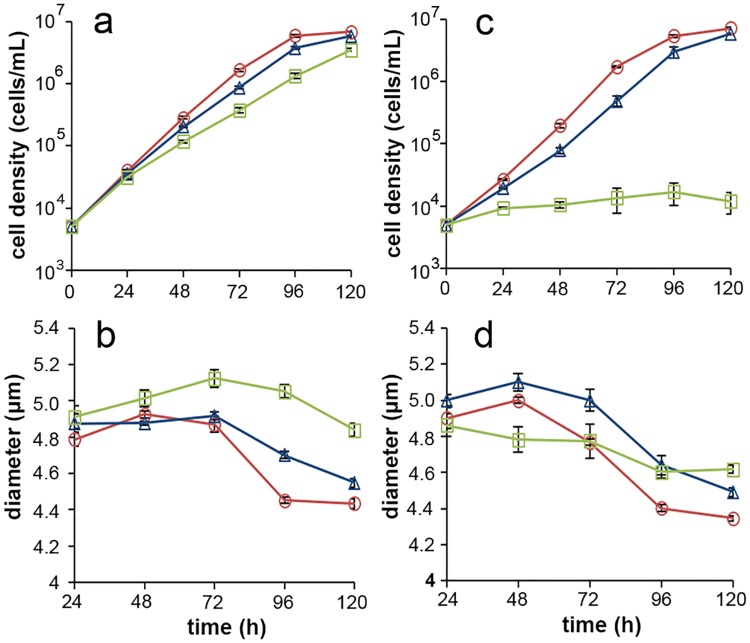
Effects of Cr(VI) and 3,5-DCP on cell density and size. *Pseudokirchneriella subcapitata* was exposed to Cr(VI) (a and b) or 3,5-DCP (b and d), and the cell density (a and c) and diameter (b and d) were measured every 24 h. In (a and b), red (open circle), control; blue (open triangle), Cr(VI) 0.15 mg/L; green (open square) 0.3 mg/L Cr(VI). In (b and d), red, control; blue, 2.0 mg/L 3,5-DCP; green 4.0 mg/L 3,5-DCP. Note that the cell sizes measured by the electronic particle counter differ from the sizes shown in Fig 1 because cell size of *P*. *subcapitata* is different in longitudinal and transvers axis. Error bars denote standard deviations with 95% confidence limits.

Along with cell density and size, we measured the frequency distributions of cell reproductive patterns (two-, four-, and eight-autospore types) under exposure to either of two typical toxicants, namely K_2_Cr_2_O_7_ and 3,5-DCP. These frequencies were measured by sampling and staining cells with DAPI every 24 h until 120 h after the start of exposure. In control culture, cell density increased exponentially until 72 h after the beginning of culture, corresponding to a specific growth rate of 1.95 day^−1^ (Fig 3a and 3c); cell density approached the stationary phase between 96 and 120 h. The mean coefficient of variation (CV) for section-by-section growth rates (0 to 24, 24 to 48, and 48 to 72 h, in the case of 72-h tests) in the control culture was 8.45%. The CV of the average specific growth rates during the whole test period in replicate control cultures was 1.17%. These values fulfilled the validity requirements for test algal species, as defined by OECD TG201 [14]. The average cell diameter increased until 48 h after the beginning of control culture (4.92–5.00 μm), but it then decreased until 96 h (approximately 4.4 μm), whereafter it stabilized or decreased only slightly (Fig 3a and 3c).

In the toxin-exposed cultures, cell densities generally declined with increasing concentration of Cr(VI) or 3,5-DCP (Fig 3a and 3c). Under 0.3 mg/L of Cr(VI) (approximately the EC_50_ value), the average cell diameter increased until 72 h after the beginning of culture (to a value greater than that in control culture) (5.12 μm) and then gradually decreased from 72 to 120 h (4.83 μm at 120 h), without the sharp decrease between 72 and 96 h seen in the control culture (Fig 3b). Under 0.15 mg/L Cr(VI), the average cell diameter was about equal to that in control culture until 72 h after the start. From 72 to 120 h it decreased but was greater than that in control culture (4.70 μm at 96h and 4.54 μm at 120h) (Fig 3b). Under 2.0 mg/L 3,5-DCP (approximately the EC_50_ value), the average cell diameter increased until 48 h after the start of culture (5.10 μm); it then decreased and was still decreasing 120 h after the start of culture (4.49 μm) (Fig 3d). Throughout the test period, the average cell diameter was larger than in the control culture (Fig 3d). Under 4.0 mg/L 3,5-DCP, cell density was arrested throughout the test period (Fig 3d), and unlike in control culture or with 2.0 mg/L 3,5-DCP, the average cell diameter did not show sharp fluctuations (4.61–4.86 μm) (Fig 3d).

**Fig 4:**
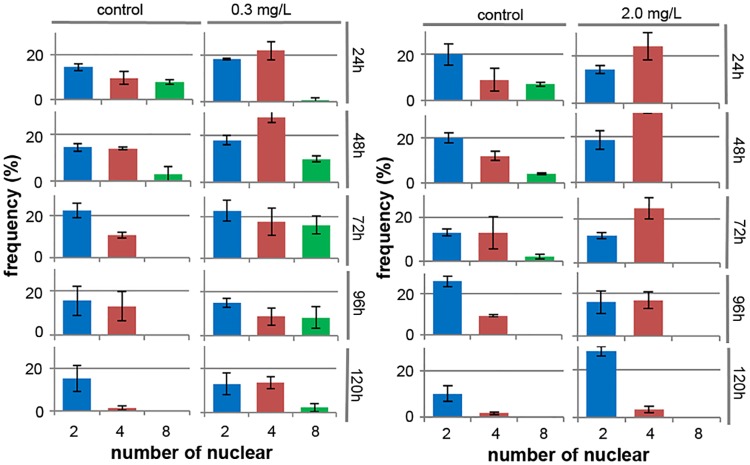
Frequency distributions of different cell reproductive patterns in *Pseudokirchneriella subcapitata* in the presence of Cr(VI) or 3,5-DCP. Observation frequencies of cells of the two-, four-, or eight-autospore type were measured by staining with DAPI every 24 h until 120 h after the start of exposure to Cr(VI) (a) or 3,5-DCP (b). Although single nuclear cells were not counted, it is equal to the remains of total frequencies of the two-, four-, or eight-autospore type. Data are means of five independent experiments. Error bars denote standard deviations with 95% confidence limits.

The frequency distributions of the different cell reproductive patterns upon exposure to Cr(VI) or 3,5-DCP varied substantially from that in control culture (Fig 4a and 4b). Eight-nucleus cells were observed with the highest frequency 24 h after the start of control culture; the frequency with which they were observed declined as culture continued, and these cells were barely observable at 72 h (Fig 4a and 4b). Between 72 and 120 h, eight-nucleus cells completely disappeared from the control culture, whereas two- or four-nucleus cells were observed in addition to single nucleus cells between 24 and 120 h and four-nuclei cells were observed at moderately high frequencies from 24 to 96 h, after which their frequencies dropped dramatically (Fig 4a and 4b). At 120 h only two-nucleus cells were observed in addition to single-nucleus cells, indicating that most of the cells in this phase were proliferating with the two-autospore type of pattern (Fig 4a and 4b). Under 0.3 mg/L of Cr (VI), the peak observation frequency of eight-nucleus cells occurred 72 h after the start of exposure; this was markedly delayed compared with that in control culture (24 h; Fig 4a). Subsequently, although their frequency gradually declined (Fig 4a), eight-nucleus cells were found even 120 h after the start of culture. Throughout culture, the total frequency of cells in the mitotic stage was much higher under 0.3 mg/L Cr (VI) than in control culture (Fig 4a).

**Fig 5:**
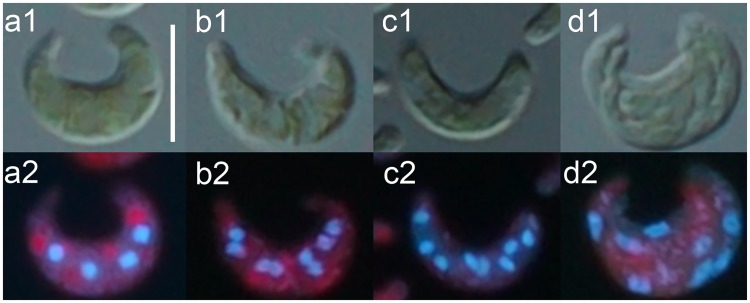
Cells of *Pseudokirchneriella subcapitata* of the eight-autospore type in the presence of Cr(VI). Cells were observed by differential interference contrast microscopy (a1-d1) or DAPI staining (a2-d2). (a), multinucleated cell with four nuclei; (b and c), multinucleated cells with eight nuclei; and (d), eight autospores in a parental cell. Scale bar in (a1), 10 μm. Scale bar in (a1) applies to (b-d).

In contrast, under 2.0 mg/L 3,5-DCP, eight-nucleus cells were never observed throughout the culture. Whereas four-nucleus cells were found with frequencies higher than those in control culture (Fig 4b). Under toxicant exposure there were no abnormalities of nuclear or cytoplasmic division in cells of the eight-autospore type (Fig 5) or those of the two- or four-autospore type (data not shown).

## Discussion

**Fig 6:**
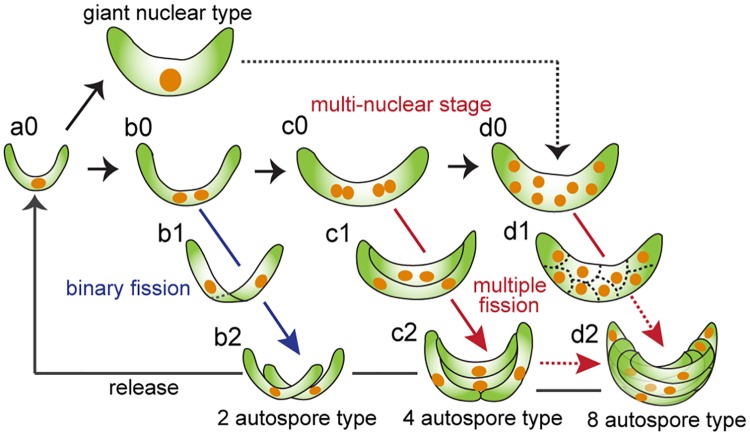
Diagrammatic representation of different cell reproductive patterns found in *Pseudokirchneriella subcapitata*. Three cell reproductive types were observed: a two-autospore type (b0-b2); a four-autospore type (c0-c2) (most frequently observed in the population in the 72 h after the start of culture), in which cytokinesis occurs by repeated binary fission after two nuclear divisions; and an eight-autospore type (d0-d2), in which autospore formation begins with separation of the cytoplasm into eight compartments, not by repeated binary fission as in the four-autospore type. The giant nucleus—type cell may also proceed to eight-autospore formation after multinucleation by three nuclear divisions. (c2) and (d2) also may occur as part of eight-autospore formation when the cell cycle restarts without the release of autospores.

Here, we report two additional reproductive patterns in *P*. *subcapitata* that form two or eight autospores, apart from the already known reproductive pattern in which four autospores are formed by multiple fission after two nuclear divisions [4] (Fig 6). In addition, by DAPI staining we revealed in detail the behaviors of nuclear and cytoplasmic division in the mitotic stage (Fig 6). The pattern of cytoplasmic division and an arrangement of autospores was different from that of other species that belong to same family (Selenastraceae) as *P*. *subcapitata* such as *Podohedriella*, *Quadrigula* and *Kirchneriella*, of which autospores were arranged either parallel or serially followed by cytoplasmic division along the longitudinal or transvers axis of the cell [23,24], whereas similar to that of *Monoraphidium* and *Ankistrodesmus* [25]: in the case of four-autospore type in *P*. *subcapitata*, cytoplasm divided with both patterns, and in the case of eight-autospore type, the autospores was arranged with complex manner (Fig 6). Moreover, we found that reproductive patterns varied substantially with the culture conditions or with exposure to either of two typical toxicants, Cr(VI) and 3,5-DCP. To our knowledge, this is the first report to evaluate the reproductive patterns of *P*. *subcapitata* in detail; these patterns changed dramatically in the presence of toxicants.

In *P*. *subcapitata*, effects of different in pH, a supply of CO_2_ and cell density on algal growth have already been studied [1]. However, it is unclear which factors influence reproductive patterns in *P*. *subcapitata*. In *Ankistrodesmus*—a genus closely related to *Pseudokirchneriella—*cell have been reported to divide by multiple fission in the exponential growth phase and by binary fission near the stationary phase, when there is a high cell density [26,27]. Also, in *Chlorella* and *Scenedesmus*, the number of daughter cells from one mother cell is influenced by various factors such as light intensity and temperature [28–33]. We consider that a *P*. *subcapitata* cell proliferates by forming more daughter cells from one mother cell by multiple fission under better culture conditions (i.e. in terms of the medium, light intensity, temperature, and cell density). This is based on our observation of the eight-autospore type relatively early in culture medium without toxicants (this assumes that the early culture conditions are better); of the four-autospore type in mainly the mid-phase of culture; and of a predominance of the two-autospore type near the stationary phase. This speculation is also supported by our finding that when cell density was suppressed by exposure to the respiration inhibitor 3,5-DCP, cells of the eight-autospore type completely disappeared (see Fig 4b). However, the results we obtained with Cr(VI) do not seem to fit this hypothesis: Cr(VI) did not suppress the appearance of cells of the eight-autospore type, although it substantially delayed the timing of their appearance compared with the ones in control culture. We consider that this phenomenon may have been due to a reduction in the Cr(VI) content of the medium—for example, because of adsorption to the cell wall, uptake into the cells, reduction from Cr(VI) to Cr(III), or some other unknown effect of Cr(VI) on the cell cycle. These results also suggest that observation of reproductive patterns in *P*. *subcapitata* may help to elucidate the reactions of different cells to toxicants.

We could not observe autospore formation by repeated binary fission in multinucleated (eight-nucleus) cells. Alternatively, we found cells with furrows around each of the eight nuclei. Hence, autospore formation in eight-nucleus cells may begin with separation of the cytoplasm into eight components (Fig 6), as occurs in the formation of the unilocular sporangia often seen in the sori of matured algae; this is different from the process seen in the four-autospore type, which divides by repeated binary fission after multiple nuclear division. There might be another possibility for eight-autospore formation: cells with four autospores might enter the next cell cycle without the release of the autospores (Fig 6). Large cells with a single giant nucleus (“giant nucleus type”) may also be involved in eight-autospore formation (Fig 6), considering the report that in *Scenedesmus armatus* and *Chlamydomonas eugametos* the number of daughter cells from a mother cell is related to cell size [34]: that is, the cells must double several times in size to divide by multiple fission. Therefore, growth is coupled to more than one checkpoint within one cell cycle, leading to multiple DNA replications and nuclear division rounds [34]. This also supports our hypothesis that the giant nucleus type is another source of eight-autospore formation in *P*. *subcapitata*.

We observed that the average cell size of *P*. *subcapitata* increased upon exposure to Cr(VI) or 3,5-DCP. This phenomenon is already well known in some algae, including *P*. *subcapitata*. Franklin et al. [5] reported that copper causes an increase in cell size in *P*. *subcapitata*. Machado and Soares [4] analyzed in detail the effects of metals (Cd, Cr, Cu, and Zn) on cell volume in *P*. *subcapitata*. Low concentrations of Cr(VI) or Cu(II) caused a decrease in cell volume, whereas high concentrations (close to the EC_50_ values) caused an increase in cell volume. Cd(II) and Zn(II) had the opposite effect: low concentrations of Cd(II) and Zn(II) caused an increase in cell volume, whereas high concentrations caused a decrease in cell volume. Similar effects of metals on cell size have been observed in other algae, such as *Scenedesmus vacuolatus*, *Chlorella* sp., *Phaeodactylum tricornutum*, and *Nitzschia closterium* [6–10]. We found here that multinucleated cells (four or eight nuclei-cells) in the mitotic stage occurred with much higher frequencies under Cr(VI) and 3,5-DCP than in control culture. As multinucleated cells in the mitotic stage are much larger than interphase cells with single nuclei (see Fig 2), we considered that the increase in average cell size under the toxicants may have resulted from accumulation of the multinucleated cells in the mitotic stage. On the other hand, in *Chlamydomonas acidophila* and *Scenedesmus quadricauda*, Cd(II) can induce increases in the number and volume of starch grains and vacuoles, resulting in an increase in cell size [35,36]. Whether this phenomenon is related to the increase in average cell size in *P*. *subcapitata* under toxicants is unclear; size measurements of individual cells, together with electron microscopic observations, would provide strong evidence to help answer this question. Bišová and Zachleder [36] revealed that at concentration of about 60 μmol/L of CdCl_2_, although DNA replication is inhibited, the cells grew to the same or lager size than did untreated cells in *Scenedesmus quadricauda*, indicating the different sensitivity of growth and reproductive events to toxicants. Our result that although the cell density was reduced by exposure to either of the two toxicants, the total frequency of occurrence of cells in the mitotic stage was much higher than in the control culture may have been caused by a delay in, or arrest of, the cell cycle in the mitotic stage by exposure to the toxicants. Machado and Soares [1] observed in *P*. *subcapitata* that exposure to a high concentration (2.13 mg/L) of Cr(VI) resulted in the accumulation of a cell population in the single-nucleus stage with larger cell size than did untreated cells, indicating that although the cell cycle was arrested before the first nuclear division, the cells grow. This is markedly different from our finding that exposure to 0.3 mg/L of Cr(VI) led to the accumulation of multinucleated cells in the mitotic stage. Probably the difference was due to the use of a higher concentration of Cr(VI) than in our study. This indicates that cell reproductive patterns in *P*. *subcapitata* also varied with the concentration of the toxicant.
